# Clinical Improvement After Treatment With IncobotulinumtoxinA (XEOMIN®) in Patients With Cervical Dystonia Resistant to Botulinum Toxin Preparations Containing Complexing Proteins

**DOI:** 10.3389/fneur.2021.636590

**Published:** 2021-02-09

**Authors:** Harald Hefter, Christian J. Hartmann, Ulrike Kahlen, Sara Samadzadeh, Dietmar Rosenthal, Marek Moll

**Affiliations:** Department of Neurology, University of Düsseldorf, Düsseldorf, Germany

**Keywords:** incobotulinumtoxinA, cervical dystonia, complexing proteins, partial secondary therapy failure, neutralizing antibodies

## Abstract

This study investigated the clinical long-term effect of incobotulinumtoxinA (incoBoNT/A) in 33 cervical dystonia (CD) patients who had developed partial secondary therapy failure (PSTF) under previous long-term botulinum toxin (BoNT) treatment. Patients were treated four times every 12 weeks with incoBoNT/A injections. Physicians assessed treatment efficacy using the Toronto Western Spasmodic Torticollis Rating Scale (TWSTRS) at the baseline visit, week 12 and 48. Patients rated quality of life of CD with the Craniocervical Dystonia Questionnaire (CDQ-24). Titres of neutralizing antibodies(NAB) were determined at start of the study and after 48 weeks. All patients had experienced significant and progressive worsening of symptoms in the last 6 months of previous BoNT treatment. Repeated incoBoNT/A injections resulted in a significant reduction in mean TWSTRS at week 12 and 48. Patients' rating of quality of life was highly correlated with TWSTRS but did not change significantly over 48 weeks. During the 48 weeks -period of incoBoNT/A treatment NAB titres decreased in 32.2%, did not change in 45.2%, and only increased in 22.6% of the patients. Thus, repeated treatment with the low dose of 200 MU incoBoNT/A over 48 weeks provided a beneficial clinical long-term effect in PSTF and did not booster titres of NAB.

## Introduction

Intramuscular injections of botulinum neurotoxin (BoNT) have become the treatment of choice for patients with cervical dystonia (CD) (1, 2). BoNT preparations are high molecular weight aggregates of the biologically active neurotoxin (a polypeptide with a 100 kDa heavy chain and a 50 kDa light chain) and complexing proteins (hemagglutinating and non-hemagglutinating) (3, 4). The BoNT/A formulation onabotulinumtoxinA (onaBoNT/A; Botox®, Allergan Inc, Irvine, USA) is composed of a 900 kD complex (so-called LL complex), the abobotulinumtoxinA formulation (aboBoNT/A; Dysport®, Ipsen Ltd., Slough, UK) is probably a mixture of 600 kD L complex and 300 kD M complex and the BoNT/B formulation rimabotulinumtoxinB (rimaBoNT/B) Myobloc™, Solstice Neurosciences Inc, San Francisco, USA and NeuroBloc®, Eisai Ltd., Hertfordshire, UK) consists of a 700 kD complex (4, 5).

Repeated BoNT injection therapy can lead to reduced responsiveness to treatment [partial secondary treatment failure (PSTF)] and to the development of neutralizing antibodies (NABs) against botulinum neurotoxin. It has been suggested that a higher content of bacterial proteins might contribute to this secondary treatment failure (6). Potential adjuvant activity of the complexing proteins is also discussed (7, 8). For instance, neutralizing antibodies had been detected in more than 17% of CD patients following onaBoNT/A treatment (9–11) before the protein content in this preparation was altered in 1998. Following the reduction of protein content, NABs were only reported in 1.2% of the patients receiving onaBoNT/A (12). For aboBoNT/A, a secondary non-responder rate of ≤ 5% and a NAB rate of >2% was found (13). In more recent cross-sectional studies prevalence of NABs was found to be even larger than 10% in patients being long-term treated over more than 10 years (14, 15).

However, additional factors to size and amount of complexing proteins must play a crucial role for the generation of resistance to a BoNT formulation, since high secondary non-responder rates of up to 44% have been observed in CD treatment with the 700 kD BoNT/B formulation already after a few injection cycles (16, 17). Most likely, the percentage of biologically inactive, but still immunologically relevant fragments of the neurotoxin may play a crucial role in the antigenicity of a BoNT formulation (4, 18).

Once complete secondary treatment failure (CSTF) has occurred and high titres of NABs have been induced, it is recommended to terminate treatment with the administered BoNT serotype (9, 19). In patients with PSTF some clinical response at week 4 may still be observed when high doses of botulinum toxin are used (20). In most patients with NAB induced PSTF a clear decrease of duration of clinical response is the first clinical sign of PSTF, the response at week 4 may persist, although relevant NAB titers have already been induced (20–22). If treatment failure has occurred after treatment with ona- or aboBoNT/A or rimaBoNT/B, the use of an alternative BoNT preparation containing complexing proteins usually does not overcome non-responsiveness (21). Therapy with different BoNT serotypes such as type B and type F may initially be successful (21, 23, 24), but will also induce antibody formation after few applications (21). To overcome antibody-induced treatment failure, extraction of NABs by plasmapheresis and immunoadsorption was successfully applied (21, 25) but was found not clinically practicable (21). Nowadays, these patients are considered to be candidates for deep brain stimulation (26).

Since July 2005, incobotulinumtoxinA (incoBoNT/A; Xeomin®, Merz Pharmaceuticals GmbH, Frankfurt, Germany) is available for the treatment of focal dystonias (27). Using an innovative purification procedure, all complexing proteins are removed resulting in a preparation containing only the pure botulinum neurotoxin (150 kD) and the lowest protein load of all available BoNT/A formulations (18, 28). Since a reduction in complexing proteins is thought to reduce the risk of NAB development and secondary non-responsiveness, this risk may be low under incoBoNT/A treatment (15, 29).

After this new BoNT/A formulation had become available, the question arose whether patients with resistance to abo- or onaBoNT/A may recapture benefit when treated with incoBoNT/A.

One might argue that incoBoNT/A administration in the treatment of CD patients with the previous PSTF will not have any clinical effect because the neurotoxin is completely unprotected against the attacks of neutralizing antibodies. On the other hand, incoBoNT/A is manufactured differently to the other BoNT/A preparations and may have a slightly different 3D-structure, which is relevant for NAB binding (4, 18). It is therefore theoretically possible that some NABs induced by abo- or onaBoNT/A do not detect and do not reduce the biological function of incoBoNT/A (30). If this were the case at least some of the patients with PSTF would respond progressively and the NAB titres would decline after switch to incoBoNT/A

We, therefore, designed the following open, prospective, non-interventional study to analyse the clinical efficacy and the development of antibody titres after four injections of the low dose of 200 MU incoBoNT/A in a cohort of 33 partial secondary non-responders to BoNT preparations containing complexing proteins.

On the basis of the current recommendations of treatment management of CD-patients with partial secondary treatment failure and antibody formation, we had to expect that patients continued to worsen and antibody titres were boostered.

## Patients and Methods

### Compliance With Ethical Standards

This open, prospective, observational, non-interventional, single center study was carried out according to the Declaration of Helsinki and Good Clinical Practice.

Informed consent was obtained from all individual participants included in the study. Local ethics committee of the Heinrich-Heine-University Duesseldorf, Germany (#4085) approval was obtained allowing to take blood samples and to determine the antibody status and publish these data in combination with anonymous clinical data of patients having given informed consent.

### Definition of Partial Secondary Treatment Failure

Criteria for partial secondary treatment failure (PSTF) were: (i) the patient had previously had a good response by at least 3 TSUI score points (31), (ii) the patient presents with a systematic worsening of CD despite dose increase and/or change of BoNT preparations containing complexing proteins. Systematic worsening was defined as an increase by at least two points over three consecutive TSUI scores each determined about 3 months after injection; (iii) the patient reports reduced efficacy for these last three consecutive injections in comparison to previous injections [for a detailed discussion of the definition of PSTF see Hefter et al. (32)].

### Patients and Intervention

The charts of all CD patients attending our botulinum toxin outpatient clinic were screened for eligibility; 55 patients presented with PSTF according to our definition (see section Definition of Partial Secondary Treatment Failure) and were informed on 4 different therapy options: (1) to participate in the present study, (2) to continue BoNT/A therapy out-side this study, (3) to cessate BoNT therapy, and (4) to undergo deep brain stimulation. Thirty-three of these patients gave informed consent to participate and were consecutively recruited. The other 22 patients decided to undergo deep brain stimulation (*n* = 20) or to stop BoNT therapy (*n* = 2).

Most (*n* = 25) of the 33 recruited patients had already previously been included in a study on treatment of *de novo* CD-patients with 500 U aboBoNT/A and had clinically been characterized very well (33). At the time of recruitment 24 patients had a main rotational component, nine a main lateral component. None of the patients suffered from a pure antecollis or antecaput (34). In 10 patients a severe additional retrocomponent was present, in 15 an additional shoulder elevation and in seven patients a moderate to severe head tremor. As described previously patients with head tremor had responded quite well (33). A second worsening with head tremor was a sensitive and objective symptom for the development of PSTF. Since 2003 CD-patients in our institution are treated according to the cap/col-concept (35) which takes into account the differences between neck and head position and movements and of the underlying activity of muscles causing these different head positions and movements (35).

After recruitment demographical and treatment-related data [date of the last two injections (T-1, T-2), the preparation used, total dose, dose per muscle, and corresponding TSUI scores] were extracted from the charts.

Patients received intramuscular injections of 200 U incoBoNT/A without EMG guidance every 12 weeks (four injection cycles = 48 weeks) according to their previous BoNT injection protocols. If a special muscle M had been treated with a dose TM, it was treated with a Xeomin® dose XM (=200U^*^TM/T) after the switch to incoBoNT/A, where T is the total dose of the previous preparation.

### Outcome Measures

#### Toronto Western Spasmodic Torticollis Rating Scale (TWSTRS)

The severity of CD was assessed by the treating physicians (UK or MM) at baseline visit T0, at week 12 (T1) and at week 48 (T4). When UK had scored the patient the first time MM analyzed the patient the next time and vice versa. Who treated the patient the first time in the study varied randomly. HH collected the data so that the scoring physician was not biased by the preceding investigation. The TWSTRS total score (range 0–85 points) (36) was used which consists of the three scores for the subscales severity (range 0–35), disability (0–30), and pain (0–20). The subscales of disability and pain are based on the patients' subjective assessments.

Since the severity of CD had worsened before therapy was switched to incoBoNT/A, patients were considered treatment responders if their scores on the TWSTRS severity subscale at week 48 had improved from baseline by ≥3 points. Patients with an improvement of more than five points were classified as very good responders. Definite non-response was present when the previous worsening continued and a further increase of three points or more was found. A TWSTRS change from baseline of no more than ±2 points was regarded as no change. Our definition of treatment response was based on the results of a previous randomized, double-blind, comparator trial between onaBoNT/A, and incoBoNT/A (27) and will be discussed in detail in section “Is the Improvement of TWSTRS Severity Score Under incoBoNT/A clinically Relevant?”.

#### Craniocervical Dystonia Questionnaire (CDQ24)

Quality of life (QoL) was rated by the patients at baseline and after 12 and 48 treatment weeks using the craniocervical dystonia questionnaire (CDQ-24), a 24-item disease-specific instrument based on the five subscales: stigma, emotional well-being, pain, activities of daily living, and social/family life (37). Patients with more than 20% improvement of CDQ24 were classified as responders, those with a worsening of more than 20% were classified as non-responders.

### Antibody Testing

Blood samples for BoNT antibody testing were collected at the start of the trial and after 48 weeks. Antibody titres were determined by an independent blinded contractor (Toxogen GmbH, Hannover, Germany) using the sensitive mouse hemidiaphragm assay (MHDA) for neutralizing antibodies (19). The upper and lower limit of neutralizing antibody detection were 10 and 0.1 mU/ml, respectively. All blood samples were analyzed at the same time following the collection of all clinical data to avoid any influence of knowing patients' antibody status according to clinical scoring procedure. One sample was lost in transport and one sample was spilled; 64 samples were analyzed.

### Statistical Analysis

The primary outcome measures of the study were the change from baseline (before first incoBoNT/A injections) to week 12, and to the end of the 4th treatment cycle at week 48 in TWSTRS severity and total score and CDQ-24. The Wilcoxon test was used to analyse non-parametrically these paired measurements. For correlations the non-parametric Spearman's rho was used. All tests were part of the commercially available statistics package SPSS (version 23; Armonk, USA).

### Results

#### Significant Worsening of CD Severity Prior to incoBoNT/A Treatment

**Figure 1:**
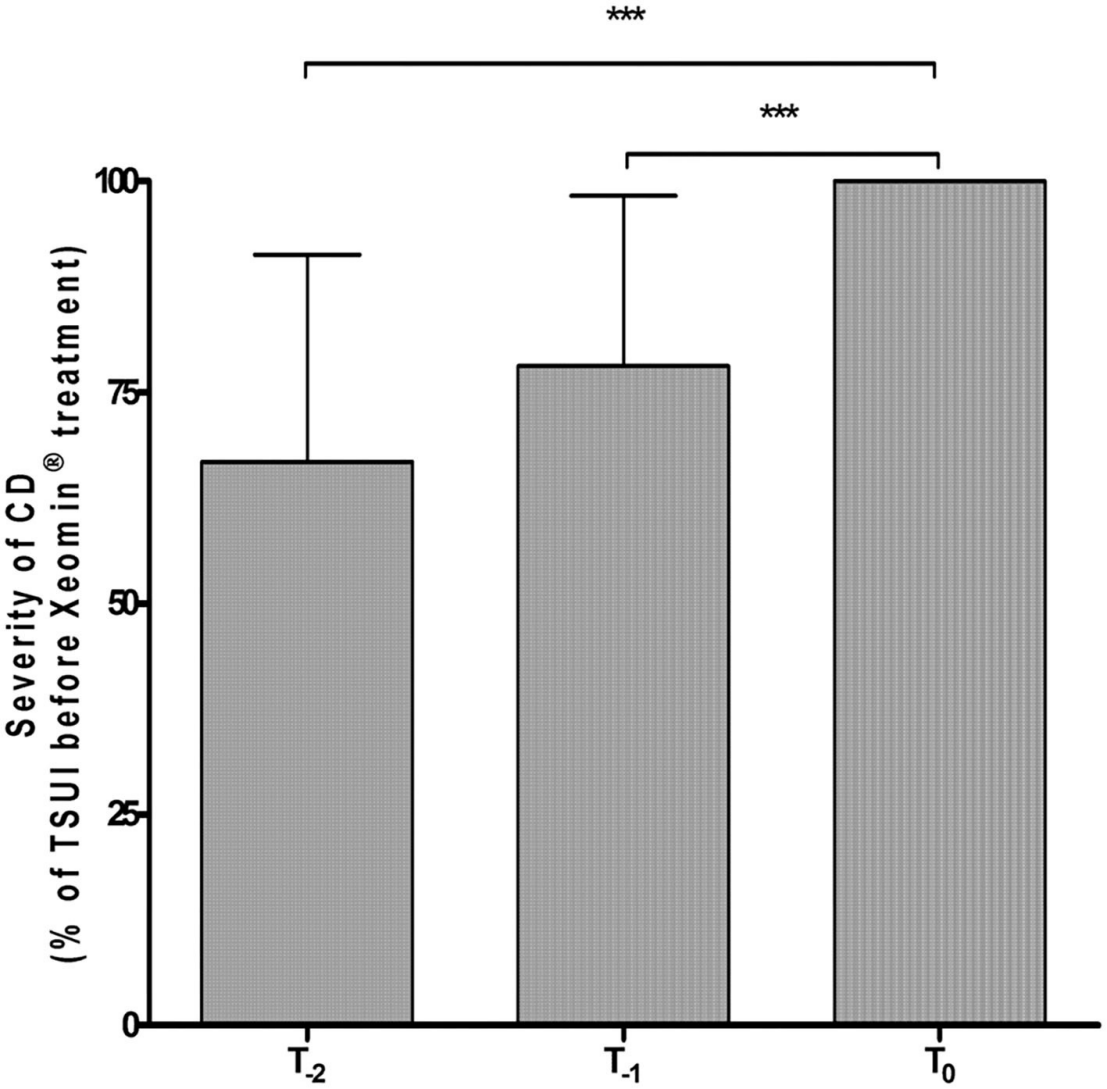
Highly significant worsening of mean normalized TSUI score during the last 6 months under previous BoNT treatment before the first incoBoNT/A injection. ***p < 0.001. T-2 = Mean normalized TSUI score before the second last BoNT injection prior to incoBoNT/A treatment; T-1 = Mean normalized TSUI score before the last BoNT injection prior to first incoBoNT/A treatment; T0 = normalized baseline TSUI score (=100%) just before first incoBoNT/A treatment. TSUI-scores at T-2 and T-1 were normalized to baseline TSUI-score at T0.

Thirty-three CD patients (17 females/16 males; mean age 56.4 ± 5.2 years) under long-term BoNT treatment presenting with partial secondary therapy failure were included in the study. At the last injection prior to study entry, six of the patients (18.2%) had received onaBoNT/A, 17 (51.5%) aboBoNT/A, and 10 (30.3%) had already been switched from a type A preparation to rimaBoNT/B. Dose ranges of the previous BoNT preparations during the last two injections were 200–300 MU onaBoNT/A, 800–1,200 MU aboBoNT/A, and 10,000–15,000 MU rimaBoNT/B. In line with our definition of PSTF, all patients experienced highly significant deterioration (*p* < 0.001) of CD severity during the last 6 months before onset of incoBoNT/A therapy (Figure 1). For each patient, TSUI-scores of the last two injections at T-2 and T-1 were normalized to patient's baseline TSUI-score at T0 (=100%). Mean normalized TSUI-scores at T-2 and T-1 were highly significantly (*p* < 0.001) lower than mean baseline TSUI-score. There was no difference in deterioration between patients previously treated with aboBoNT/A, onaBoNT/A, or rimaBoNT/B.

#### Significant Improvement of CD Severity After incoBoNT/A Treatment

**Figure 2:**
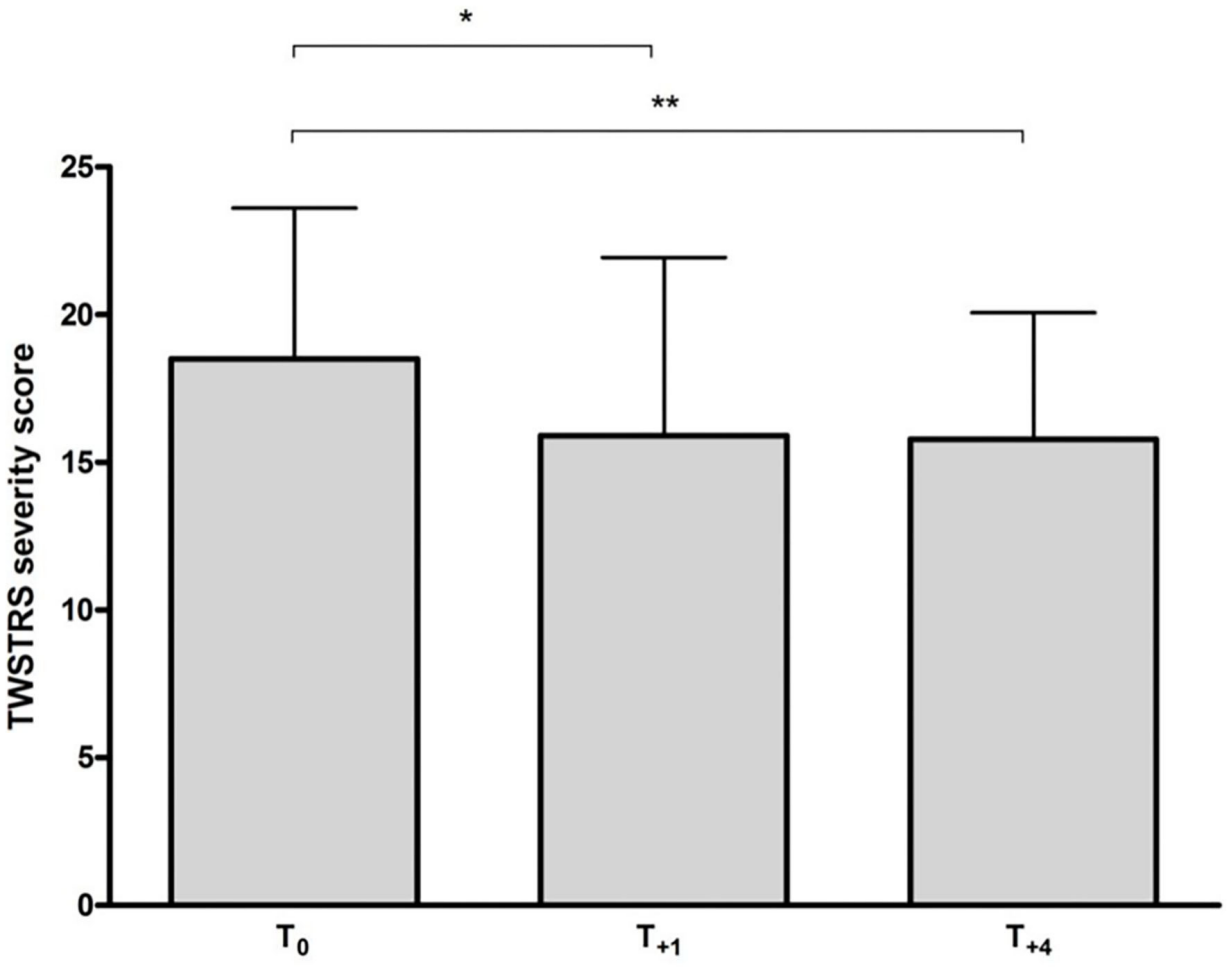
Mean (+*SD*) TWSTRS severity subscore at baseline and following incoBoNT/A injections. *p < 0.05; **p < 0.01. T0 = mean baseline TWSTRS just before first incoBoNT/A treatment; T+1 = mean TWSTRS after 12 weeks just before second incoBoNT/A treatment; T+4 = mean TWSTRS 12 weeks after 4th incoBoNT/A treatment.

**Figure 3:**
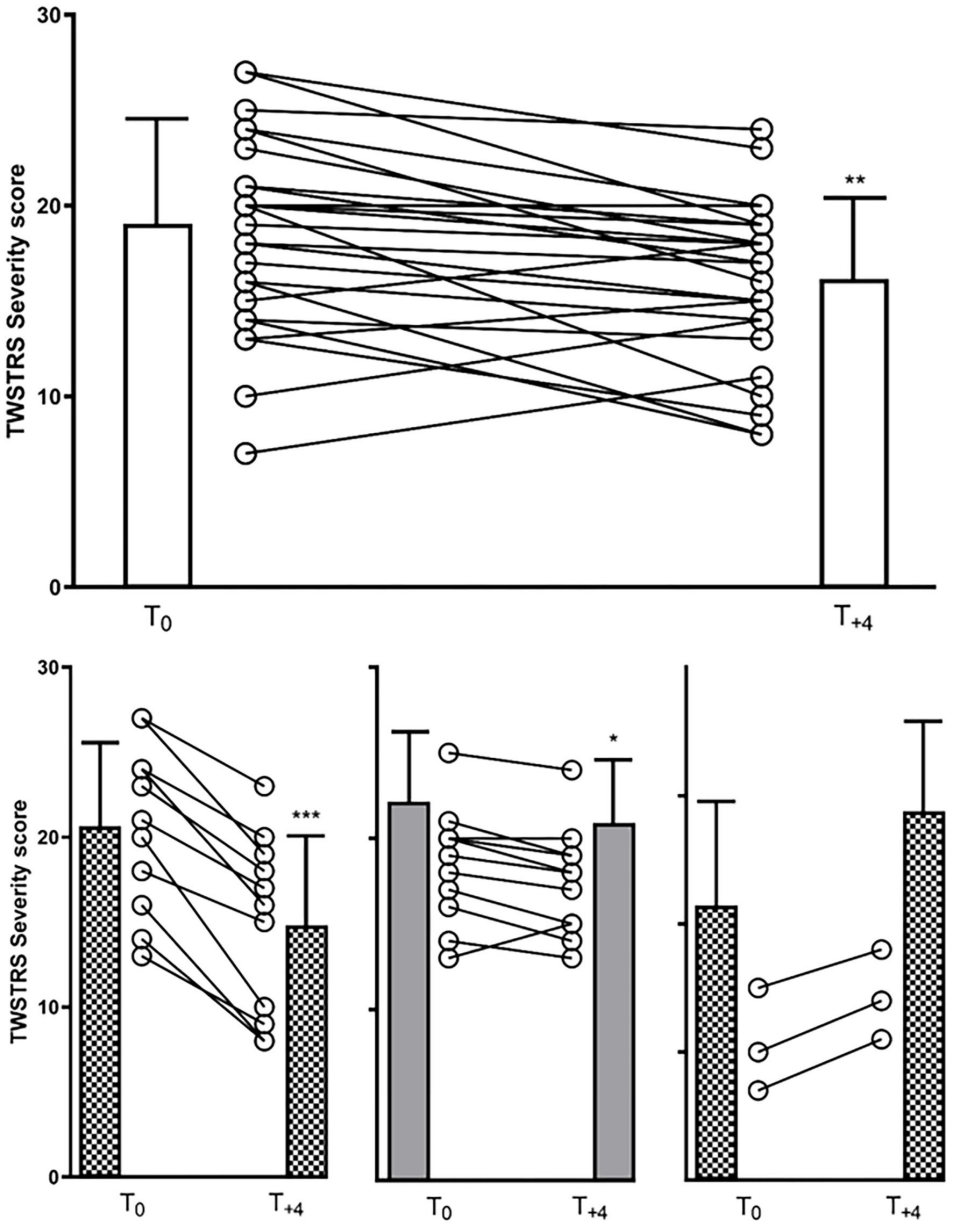
(upper part): TWSTRS severity subscore of the entire cohort at baseline and week 48 (12 weeks after the last of four incoBoNT/A injections). Mean values with corresponding SDs and individual data of 25 patients are shown. T0 = baseline TWSTRS severity score just before first incoBoNT/A treatment; T+4 = TWSTRS severity score 12 weeks after 4th incoBoNT/A treatment. (lower part): Similar presentation as in the upper part for the responder group (left side), the possible responder group (middle part) and the non-responder group (right part). *P < 0.05; **P < 0.01; ***P < 0.001.

A significant reduction in TWSTRS severity subscore compared to baseline was observed at week 12 (*p* < 0.05) and at week 48 (after 4 incoBoNT/A injections; *p* < 0.01; Figure 2). Because of incomplete data for the TWSTRS score, a direct comparison between baseline and week 48-TWSTRS severity score was only possible in 25 patients. Compared to baseline score TWSTRS severity subscore decreased and improved in 20 patients (=80%), did not change in one patient (4%) and increased in four patients (16%). Eleven of the patients were definite responders (44%) after 48 weeks, only three patients were definite non-responders (12%) and possible responders (no clear-cut change: ±2 points) were further 11 patients (44%). Changes of more than five points were seen in five patients (very good responders: 25%). The individual development in TWSTRS severity subscores is illustrated for the entire cohort in the upper part of Figure 3 and for the three responder groups in the lower part of Figure 3. Improvements were also observed at week 48 for the TWSTRS subscale pain, and for total TWSTRS; however, they failed to reach significance (Table 1).

**Table 1:** Mean (±*SD*) TWSTRS scores at week 12 and 48 of the study period.

**TWSTRS**	**Baseline**	**Week 12**	*P*-value^*^	**Week 48**	*P*-value^*^
Total score	36.0 ± 12.6	35.4 ± 12.4	ns	33.8 ± 12.6	ns
Severity score	18.1 ± 5.0	16.3 ± 5.6	<0.05	15.6 ± 4.6	<0.01
Disability score	11.7 ± 5.5	12.0 ± 5.0	ns	13.2 ± 5.5	ns
Pain score	6.2 ± 5.1	7.2 ± 6.1	ns	5.0 ± 5.4	ns

* *Compared to baseline, non-parametric testing*.

*ns, not significant; TWSTRS, Toronto Western Spasmodic Torticollis Rating Scale*.

No correlation was found between clinical response to incoBoNT/A after 12 and 48 weeks with the previous total dose or the previous duration of treatment. For sake of comparison and simplicity inco- and onaBoNT/A doses were kept constant, aboBoNT/A doses were divided by 3 and rimaBoNT/B doses by 30 following a European consensus recommendation (38) well-knowing that these conversion ratios may vary from study to study and from muscle to muscle [for an overview see (39, 40)].

#### Only Little Changes in Quality of Life During incoBoNT/A Treatment

Median CDQ-24 scores also improved from baseline to week 48 but failed to reach the significance level of 5% (Table 1). Eight of the 33 patients (24.2%) reported a 20% improvement, whereas only four patients (12.1%) considered their QoL worsened by 20% or more; in all other patients (63.7%), changes were smaller than ±20%. At week 12 patients' subjective rating of quality of life (CDQ24) at baseline was just below significance for a correlation with the physicians' TWSTRS severity ratings (*r* = 0.3694; *p* = 0.058). However, there was a highly significant correlation between the CDQ24 with the total TWSTRS, when pain and disability subscores were added to the severity subscore (*r* = 0.5792; *p* < 0.001). At week 48 no correlation between CDQ24 and total TWSTRS was found (*r* = 0.2563; *p* > 0.05).

#### Development of Antibody Titres During incoBoNT/A Treatment

Information on neutralizing antibody titers at baseline was available for 32 of our 33 CD patients with PSTF: 25 patients tested positive for the presence of neutralizing antibodies, seven patients tested negative. One of the post-incoBoNT/A samples of the patients testing negative was spilled; therefore, 31 pre/post incoBoNT/A comparisons were available (Table 2).

**Table 2:** Development of NAB titres in 31 patients before and after incoBoNT/A.

**Decreasing titres (Pat.)**	**Before**	**After**	**Equal titres (Pat.)**	**Before**	**After**
1	10	7	1	10	10
2	7	2	2	10	10
3	6	1	3	10	10
4	2	0.5	4	10	10
5	2	1	5	1	1
6	1	neg	6	1	1
7	1	0.5	7	0.4	0.4
8	0.8	neg	8	0.4	0.4
9	0.4	neg			
10	0.4	neg			
**Negative titres (Pat.)**	**Before**	**After**	**Increasing titres (Pat.)**	**Before**	**After**
1	neg	neg	1	5	10
2	neg	neg	2	3	10
3	neg	Neg	3	1	3
4	neg	neg	4	0.4	2
5	neg	Neg	5	0.4	0.8
6	neg	Neg	6	0.4	0.6
			7	0.4	0.6

All patients testing negative at baseline remained negative (6/31 = 19.4%; Table 2). Of the 25 CD patients testing positive at baseline, 10 patients (40%) had decreased antibody titers after 48 weeks (Table 2), and titers remained constant in eight patients (32%; Table 2); boostering occurred only in seven patients (28%; Table 2). Overall, an increase in NAB titres was only detected in seven of the 31 patients with available pre/post comparison data (22.6%); in all other 24 patients (77.4%) titres either declined or remained constant. Titers remained high in four patients who presented with an initially high titer. In four patients with a positive assay, the test was negative after four cycles of incoBoNT/A treatment (Table 2).

No correlation was found between initial NAB titers and the changes of TWSTRS severity scores over 48 weeks of incoBoNT/A treatment and between changes of NAB titers and changes of TWSTRS severity scores after 48 weeks. There was a non-significant trend that higher scores were associated with higher NAB titers at baseline, but not at week 48. None of the six patients testing negative at baseline and during the study (Table 2) was classified a responder.

## Discussion

The present study shows that injection therapy with a standard dose of 200 MU incoBoNT/A over a 48-week period provides a significant, beneficial clinical long-term effect in a cohort of CD patients (Figure 2), who had continuously worsened under abo- or onaBoNT/A or rimaBoNT/B pre-treatment.

### Is the Improvement of TWSTRS Severity Score Under incoBoNT/Aclinically Relevant?

In contrast to other studies on the efficacy of single BoNT injections for CD treatment, where the primary efficacy analysis is assessed at week 4 following treatment (2, 27, 41), efficacy analysis in the present study was performed at the end of an injection cycle just before the next injection cycle was started. This is important to keep in mind since in patients with NAB-induced PSTF the 4-weeks effect may be preserved whereas the duration of the efficacy has already declined (21). By means of the method used here, improvement can only be detected when the effect of an incoBoNT/A injection lasts longer than 12 weeks. Therefore, the significant improvement in the present study, therefore, does not reflect a transient 4-weeks effect, but indicates a permanent improvement during the entire injection cycle lasting months. Thus, efficacy analysis in the present study is highly conservative.

Our data compare well to a randomized, double-blind, comparator trial between onaBoNT/A and incoBoNT/A (27) in patients responding well to onaBoNT/A. Mean improvement of the TWSTRS-severity subscore 16 weeks after either incoBoNT/A or onaBoNT/A injection was significant from baseline for both BoNT/A preparations without difference between them. The mean change in TWSTRS severity score at week 4 was in the order of−6 which decreased to−1.8 at week 16. Using our responder criterion of ≥3 points improvement in TWSTRS severity score at week 12, the corresponding responder rates at week 12 for incoBoNT/A and onaBoNT/A injections in the comparator trial can be estimated to be close to 50% (assuming a linear decrease of efficacy from week 4 to 16). In the present study, incoBoNT/A treatment of patients with partial secondary treatment failure to BoNT treatment resulted in a responder rate around 44%. This also compared well to a double-blind, randomized, controlled trial on efficacy and safety of treatment of *de novo* and well-responding CD-patients with 500 unit aboBoNT/A (42). Responder rates in the Dysport®-arm were 30/35 (=86%) at week 4, 26/35 (=74%) at week 8 and 2/35 (=5.7%) at week 16 (42). Linear interpolation to estimate the responder rate at week 16 yields 14/35 (=40%). This is also close to responder rates observed when CD patients unresponsive to BoNT/A were switched to treatment with BoNT/B (24).

When evaluating the relevance of clinical improvement with incoBoNT/A, we have to take into account that our patients experienced a highly significant deterioration during the last 6 months before the switch to incoBoNT/A treatment. Initiation of incoBoNT/A treatment did not only stop this deterioration but unexpectedly initiated a slowly progressive improvement. This improvement is not due to a large injection dose of incoBoNT/A since the dose of 200 MU incoBoNT/A is low compared to the doses of BoNT/A or BoNT/B used before the switch (see Results section Significant Worsening of CD Severity Prior to incoBoNT/A Treatment).

Some of the patients who had previously been switched to BoNT/B after PSTF to BoNT/A, had expected a larger incoBoNT/A effect and were disappointed. They tended to underestimate the effect of the switch to incoBoNT/A. On average, there was no significant change in quality of life as measured by the CDQ24. However, at least three patients reported that the effect of incoBoNT/A injections was close to the effect experienced when they had received BoNT/A injections for the first time. These patients probably overestimated the incoBoNT/A effect because of the preceding deterioration.

Similar to a large study in *de novo* CD patients correlating TSUI and CDQ24 scores (43), the correlation between TWSTRS severity subscore and CDQ24 in the present study was only weak. A much better correlation was found between total TWSTRS and CDQ24, since both scales contain items asking about pain and everyday life activities. The items in the CDQ24 addressing stigmatization correlated best with severity scores. This was also the case in the above-mentioned study (43).

### Development of Neutralizing Antibodies Under incoBoNT/A Treatment

The present results indicate that incoBoNT/A injections may be clinically effective without boostering NAB levels. In most of the patients (>77%), NAB titres did not increase despite the injection of the pure neurotoxin type A. Furthermore, titers declined as rapidly under incoBoNT/A injections as after cessation of any BoNT/A treatment (30). Doses of 200 MU incoBoNT/A with a protein load of 0.8 ng thus seem to lie close to the detection limit of the human immune system (30).

Our results also support that there is no simple relationship between NAB titers and clinical outcome in CD patients. The comparison of TWSTRS severity score and NAB titers did not show any correlation before or after incoBoNT/A treatment and no correlation between changes in both parameters. This is in full agreement with Lange et al. (44) who suggested that there is little or no relation between clinical data and NAB titers. To our experience, the paralysis time which is the direct outcome measure of the MHDA yields better correlations with clinical data than the derived NAB titers (45).

So far it is not clear why in some patients with PSTF the MHDA does not detect neutralizing antibodies. Compared to other studies analyzing antibodies in patients with PSTF (13, 44), the number of patients without detectable NABs in the MHDA was rather low (7/32 <22%). These patients did not respond better to incoBoNT/A than most of the other patients. On the other hand, NAB titers declined below the detection limit in four patients. These patients had developed PSTF under ona- or abo BoNT/A treatment with positive NAB testing but responded well to incoBoNT/A and became negative in the MHDA test after 48 weeks. This underlines how complex the problem of responsiveness to different BoNT/A preparations and the mechanisms of NAB induction in BoNT treatment of dystonias are.

Clinical changes may precede changes in NAB titers considerably. It has been shown that high antibody titers take years to decline and long-lasting treatment failure is common (20, 21, 30). Therefore, the development of neutralizing antibodies should be avoided from the very beginning. Animal experiments might help to carefully analyse the temporal course of antibody induction and changes of efficacy (46), but their interpretation is of limited value due to species differences.

### Speculations on Possible Reasons for the incoBoNT/A Effect and Lack of Correlation With Antibody Titres

It has been reported that the application of higher doses and EMG guidance may lead to clinical improvement in patients with PSTF (47). Patients in the present study were treated with 200 MU incoBoNT/A which is fairly low compared to the doses patients received prior to this study. Thus, PSTF was not overcome in the present study by means of high incoBoNT/A doses. Furthermore, patients received incoBoNT/A injections without EMG guidance following the same injection protocols as used for their previous BoNT injections. There is no reason to assume that incoBoNT/A was administered more precisely than the other BoNT formulations.

To explain the effect of incoBoNT/A treatment, one has to take into account the differences in the various BoNT preparations regarding neutralizing antibody induction. IncoBoNT/A does not contain complexing proteins. It has been reported that components of the BoNT/B haemagglutinin complex stimulate interleukin 6 production and probably enhance antibody production against the neurotoxin (7). This may also be the case for BoNT/A complexing proteins (8). Haemagglutinins act as lectins with high specificity to galactose-containing glycoproteins of glycolipids (48). Lectins are known to function as immune adjuvants. The cell-binding subunit of ricin, for example, stimulates the antibody production against a virus antigen (49). An additional factor influencing the immune response could be flagellin which was identified as a protein component of the abobotulinumtoxinA bulk toxin (50). Flagellin interacts with Toll-like Receptor 5 (TLR5) initiating an innate immune response (51) and is known to be an immunological adjuvant (52). Because of the reduced bacterial protein content of incoBoNT/A, there seems to be less sensitization of the human immune system with the pure neurotoxin than with the entire BoNT complex (4). Furthermore, because of a new purification process used for incoBoNT/A, the relation between intact (biologically active) and damaged (biologically inactive, but still immunizing) neurotoxin A is more favorable for incoBoNT/A than for the other BoNT/A preparations (18).

OnaBoNT/A, aboBoNT/A, and incoBoNT/A are manufactured quite differently. Differences in purification and vacuum extraction may have an impact on the 3D-structure of the highly complex botulinum neurotoxin molecule. But it is this 3D structure which is relevant for antibody formation and binding. If therapy failure in a patient is mediated by a monoclonal antibody-induced by abo- or onaBoNT/A which does not detect incoBoNT/A, this patient will respond as a *de novo*-patient to incoBoNT/A. IncoBoNT/A injections may, therefore, be clinically effective without boostering NAB levels during long-term treatment. In clinical practice, the spectrum of NABs in a patient whether it consists only of a monoclonal AB or contains polyclonal ABs is usually not known. Furthermore, the efficacy of a human antibody in reducing the biological function of BoNT may be different in a human being compared to the MHDA and may be a general reason why MHDA titres do not correlate well with the effect in clinical practice.

## Conclusion

The present study provides evidence that incoBoNT/A is clinically effective in the long-term treatment of CD patients who had become poorly responsive to other BoNT preparations. The present clinical data in combination with NAB measurements showing continuous improvements in CD severity as well as non-increase of NAB titers following repeated incoBoNT/A injections in the majority of CD-patients with PSTF indicate low antigenicity for incoBoNT/A. This should be further explored in a multicentre, prospective study monitoring clinical outcome as well as antibody titers before and during incoBoNT/A treatment. Confirmation of low incoBoNT/A antigenicity might lead to changes in the way CD patients are treated. Shorter intervals and higher doses could be used—a further major step in the improvement of botulinum toxin injection therapy.

## Data Availability Statement

The raw data supporting the conclusions of this article will be made available by the authors, without undue reservation.

## Ethics Statement

The studies involving human participants were reviewed and approved by Local ethics committee of the Heinrich-Heine-University Duesseldorf, Germany (#4085). The patients/participants provided their written informed consent to participate in this study.

## Author Contributions

HH designed the present study. UK, SS, MM, and CH treated the patients, determined the scores, and took blood samples for antibody testing. HH, SS, CH, and DR analyzed the results. All authors reviewed the manuscript.

## Conflict of Interest

The research work of HH has been supported by the private Inge-Diesbach-Stiftung. This allowed to pay for the antibody testing. Otherwise this support did not have any influence on the present study. The remaining authors declare that the research was conducted in the absence of any commercial or financial relationships that could be construed as a potential conflict of interest.
